# 4-(6-Quinolyl­oxymeth­yl)benzonitrile

**DOI:** 10.1107/S1600536809016560

**Published:** 2009-05-14

**Authors:** Min Min Zhao, Yong Hua Li, De Hong Wu, Qing Wan

**Affiliations:** a Ordered Matter Science Research Center, College of Chemistry and Chemical Engineering, Southeast University, Nanjing 211189, People’s Republic of China

## Abstract

The title compound, C_17_H_12_N_2_O, was synthesized by an ether synthesis from quinolin-6-ol and 4-(bromo­meth­yl)benzonitrile. The phenyl ring of the benzonitrile group makes a dihedral angle of 47.52 (6)° with the plane of the quinoline fragment. The crystal structure is stabilized by inter­molecular C—H⋯π inter­actions between a benzene H atom of the benzonitrile group and the benzene ring of the quinoline fragment. In addition, the crystal structure also exhibits a weak inter­molecular C—H⋯N hydrogen bond.

## Related literature

For general background to nitrile compounds, see: Jin *et al.* (1994 ▶); Brewis *et al.* (2003 ▶). For related structures, see: Fu & Zhao (2007 ▶); Zhao (2008 ▶).
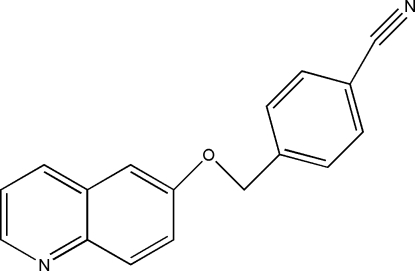


## Experimental

### 

#### Crystal data

C_17_H_12_N_2_O
*M* *_r_* = 260.29
Monoclinic, 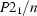

*a* = 9.466 (2) Å
*b* = 13.078 (3) Å
*c* = 10.857 (2) Å
β = 90.81 (3)°
*V* = 1343.9 (5) Å^3^
*Z* = 4
Mo *K*α radiation
μ = 0.08 mm^−1^
*T* = 293 K
0.30 × 0.26 × 0.24 mm

#### Data collection

Rigaku SCXmini diffractometer
Absorption correction: multi-scan (*CrystalClear*; Rigaku, 2005 ▶) *T*_min_ = 0.976, *T*_max_ = 0.981
12007 measured reflections
2622 independent reflections
1956 reflections with *I* > 2σ(*I*)
*R*_int_ = 0.054

#### Refinement

*R*[*F* ^2^ > 2σ(*F* ^2^)] = 0.049
*wR*(*F* ^2^) = 0.120
*S* = 1.06
2622 reflections
182 parameters
H-atom parameters constrained
Δρ_max_ = 0.15 e Å^−3^
Δρ_min_ = −0.13 e Å^−3^

### 

Data collection: *CrystalClear* (Rigaku, 2005 ▶); cell refinement: *CrystalClear*; data reduction: *CrystalClear*; program(s) used to solve structure: *SHELXS97* (Sheldrick, 2008 ▶); program(s) used to refine structure: *SHELXL97* (Sheldrick, 2008 ▶); molecular graphics: *SHELXTL/PC* (Sheldrick, 2008 ▶); software used to prepare material for publication: *SHELXTL/PC*.

## Supplementary Material

Crystal structure: contains datablocks I, global. DOI: 10.1107/S1600536809016560/lx2099sup1.cif

Structure factors: contains datablocks I. DOI: 10.1107/S1600536809016560/lx2099Isup2.hkl

Additional supplementary materials:  crystallographic information; 3D view; checkCIF report

## Figures and Tables

**Table 1:** Hydrogen-bond geometry (Å, °)

*D*—H⋯*A*	*D*—H	H⋯*A*	*D*⋯*A*	*D*—H⋯*A*
C12—H12⋯*Cg*^i^	0.93	2.83	3.613 (2)	142
C13—H13⋯N1^ii^	0.93	2.60	3.398 (2)	145

Symmetry codes: (i) 
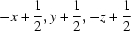
; (ii) 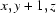. *Cg* is the centroid of the C1–C4/C8/C9 benzene ring.

**Fig. 1.**
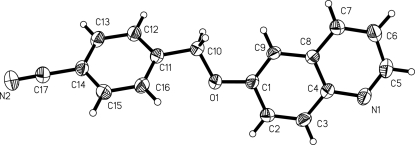
The molecular structure of the title compound with the atom numbering scheme. Displacement ellipsoids are drawn at the 30% probability level. H atoms are presented as a small spheres of arbitrary radius.

**Fig. 2.**
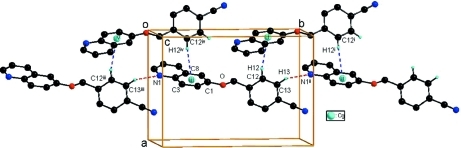
The C—H···π and C—H···N interactions (dotted lines) in the title compound. Cg denotes the ring centroid. [Symmetry codes: (i) -x + 1/2, y + 1/2, -z + 1/2; (ii) x, y + 1, z; (iii) x, y - 1, z; (iv) -x + 1/2, y - 1/2, -z + 1/2.]

## supplementary crystallographic information

### Comment

The synthesis of new azoles has been a very active area of research and one important aspect has been the incorporation of functional units. Nitrile derivatives have found many industrial applications. For example, phthalonitriles have been used as starting materials for phthalocyanines (Jin *et al.*, 1994), which are important components for dyes, pigments, gas sensors, optical limiters and liquid crystals, and which are also used in medicine, as singlet oxygen photosensitisers for photodynamic therapy (PDT; Brewis *et al.*, 2003). Recently, we have reported a few benzonitrile compounds (Fu & Zhao, 2007; Zhao, 2008). As an extension of our work on the structural characterization, Here we present the synthesis and crystal structure of the title compound 4-[(quinolin-6-yloxy)methyl]benzonitrile (Fig. 1).

The phenyl ring (C11–C16) make a dihedral angle of 47.44 (1)° with the plane of the quinoline fragment. The molecular packing (Fig. 2) is stabilized by intermolecular C—H···π interactions between the benzene H atom of benzonitrile group and the benzene ring of the quinoline fragment from an adjacent molecule, with a C12—H12···Cg^i^ separation of 2.83 Å (Fig. 2 and Table 1; Cg is the centroid of the C1–C4/C8/C9 benzene ring, symmetry code as in Fig. 2). Additionally, a weak intermolecular C—H···N hydrogen bond in the structure is observed (Fig. 2 and Table 1).

### Experimental

Quinolin-6-ol (1 g, 0.0069 mol) was added to a solution of sodium hydroxide (0.276 g, 0.0069 mol) in 15 ml of methanol and stirred for one hour. Then 4-(bromomethyl)benzonitrile (1.352 g, 0.0069 mol) was added to the above solution. The mixture was stirred at room temperature for 1 d. The title compound was isolated using column chromatography (petroleum ether:ethyl acetate = 2:1). Single crystals suitable for X-ray diffraction analysis were obtained from slow evaporation of ethyl acetate and tetrahydrofuran solution.

### Refinement

All the C—H H atoms were calculated geometrically and with C—H distances ranging from 0.93 to 0.97 Å and were allowed to ride on the C and O atoms to which they are bonded. With which *U*_iso_(H) = 1.2Ueq(C).

### Crystal data

**Table:**

C_17_H_12_N_2_O	*F*(000) = 544
*M**_r_* = 260.29	*D*_x_ = 1.286 Mg m^−^^3^
Monoclinic, *P*2_1_/*n*	Mo *K*α radiation, λ = 0.71073 Å
Hall symbol: -P 2yn	Cell parameters from 10916 reflections
*a* = 9.466 (2) Å	θ = 6.2–55.5°
*b* = 13.078 (3) Å	µ = 0.08 mm^−^^1^
*c* = 10.857 (2) Å	*T* = 293 K
β = 90.81 (3)°	Prism, colourless
*V* = 1343.9 (5) Å^3^	0.30 × 0.26 × 0.24 mm
*Z* = 4	

### Data collection

**Table:**

Rigaku SCXmini diffractometer	2622 independent reflections
Radiation source: fine-focus sealed tube	1956 reflections with *I* > 2σ(*I*)
graphite	*R*_int_ = 0.054
Detector resolution: 13.6612 pixels mm^-1^	θ_max_ = 26.0°, θ_min_ = 3.1°
ω scans	*h* = −11→11
Absorption correction: multi-scan (*CrystalClear*; Rigaku, 2005)	*k* = −16→16
*T*_min_ = 0.976, *T*_max_ = 0.981	*l* = −13→13
12007 measured reflections	

### Refinement

**Table:**

Refinement on *F*^2^	Secondary atom site location: difference Fourier map
Least-squares matrix: full	Hydrogen site location: difference Fourier map
*R*[*F*^2^ > 2σ(*F*^2^)] = 0.049	H-atom parameters constrained
*wR*(*F*^2^) = 0.120	*w* = 1/[σ^2^(*F*_o_^2^) + (0.050*P*)^2^ + 0.1913*P*] where *P* = (*F*_o_^2^ + 2*F*_c_^2^)/3
*S* = 1.06	(Δ/σ)_max_ < 0.001
2622 reflections	Δρ_max_ = 0.14 e Å^−^^3^
182 parameters	Δρ_min_ = −0.13 e Å^−^^3^
0 restraints	Extinction correction: *SHELXL97* (Sheldrick, 2008), Fc^*^=kFc[1+0.001xFc^2^λ^3^/sin(2θ)]^-1/4^
Primary atom site location: structure-invariant direct methods	Extinction coefficient: 0.018 (4)

### Special details

**Table:**

Geometry. All esds (except the esd in the dihedral angle between two l.s. planes) are estimated using the full covariance matrix. The cell esds are taken into account individually in the estimation of esds in distances, angles and torsion angles; correlations between esds in cell parameters are only used when they are defined by crystal symmetry. An approximate (isotropic) treatment of cell esds is used for estimating esds involving l.s. planes.
Refinement. Refinement of F^2^ against ALL reflections. The weighted R-factor wR and goodness of fit S are based on F^2^, conventional R-factors R are based on F, with F set to zero for negative F^2^. The threshold expression of F^2^ > 2sigma(F^2^) is used only for calculating R-factors(gt) etc. and is not relevant to the choice of reflections for refinement. R-factors based on F^2^ are statistically about twice as large as those based on F, and R- factors based on ALL data will be even larger.

### Fractional atomic coordinates and isotropic or equivalent isotropic displacement parameters (Å^2^)

**Table:**

	*x*	*y*	*z*	*U*_iso_*/*U*_eq_	
O1	0.48772 (13)	0.46617 (8)	0.29375 (10)	0.0598 (4)	
N1	0.40388 (17)	0.05815 (11)	0.18437 (15)	0.0620 (4)	
N2	0.7214 (2)	0.99565 (14)	0.44097 (18)	0.0841 (6)	
C1	0.46122 (17)	0.36816 (12)	0.25614 (16)	0.0484 (4)	
C2	0.49253 (18)	0.29346 (13)	0.34606 (16)	0.0538 (5)	
H2	0.5251	0.3134	0.4236	0.065*	
C3	0.47550 (19)	0.19262 (13)	0.32018 (16)	0.0547 (5)	
H3	0.4984	0.1441	0.3798	0.066*	
C4	0.42364 (17)	0.16054 (12)	0.20416 (16)	0.0478 (4)	
C5	0.3504 (2)	0.03171 (15)	0.07708 (19)	0.0701 (6)	
H5	0.3356	−0.0376	0.0624	0.084*	
C6	0.3139 (2)	0.10024 (15)	−0.01687 (19)	0.0691 (6)	
H6	0.2764	0.0765	−0.0912	0.083*	
C7	0.33370 (19)	0.20151 (13)	0.00168 (16)	0.0570 (5)	
H7	0.3103	0.2482	−0.0599	0.068*	
C8	0.39011 (16)	0.23550 (12)	0.11523 (15)	0.0451 (4)	
C9	0.40997 (17)	0.34014 (12)	0.14319 (15)	0.0474 (4)	
H9	0.3881	0.3898	0.0846	0.057*	
C10	0.45964 (19)	0.54615 (12)	0.20742 (16)	0.0526 (4)	
H10A	0.3588	0.5517	0.1918	0.063*	
H10B	0.5056	0.5316	0.1301	0.063*	
C11	0.51542 (17)	0.64433 (12)	0.26109 (15)	0.0478 (4)	
C12	0.43628 (18)	0.73295 (12)	0.25445 (17)	0.0531 (5)	
H12	0.3466	0.7310	0.2185	0.064*	
C13	0.48752 (18)	0.82412 (13)	0.30002 (17)	0.0552 (5)	
H13	0.4330	0.8831	0.2948	0.066*	
C14	0.62108 (18)	0.82714 (12)	0.35366 (15)	0.0494 (4)	
C15	0.70261 (19)	0.73897 (13)	0.35993 (17)	0.0576 (5)	
H15	0.7927	0.7410	0.3951	0.069*	
C16	0.64942 (19)	0.64883 (13)	0.31393 (17)	0.0574 (5)	
H16	0.7042	0.5899	0.3183	0.069*	
C17	0.6764 (2)	0.92132 (15)	0.40205 (17)	0.0607 (5)	

### Atomic displacement parameters (Å^2^)

**Table:**

	*U*^11^	*U*^22^	*U*^33^	*U*^12^	*U*^13^	*U*^23^
O1	0.0811 (9)	0.0411 (7)	0.0567 (8)	−0.0085 (6)	−0.0115 (6)	0.0033 (5)
N1	0.0743 (11)	0.0411 (9)	0.0705 (11)	0.0007 (7)	0.0028 (8)	0.0007 (7)
N2	0.0966 (13)	0.0606 (11)	0.0946 (13)	−0.0189 (10)	−0.0163 (10)	−0.0113 (10)
C1	0.0489 (9)	0.0409 (9)	0.0551 (10)	−0.0042 (7)	−0.0015 (8)	0.0030 (8)
C2	0.0596 (11)	0.0524 (11)	0.0494 (10)	−0.0030 (8)	−0.0063 (8)	0.0048 (8)
C3	0.0610 (11)	0.0479 (11)	0.0553 (11)	0.0019 (8)	−0.0030 (8)	0.0128 (8)
C4	0.0455 (9)	0.0412 (10)	0.0569 (11)	0.0011 (7)	0.0043 (8)	0.0037 (8)
C5	0.0847 (15)	0.0449 (11)	0.0808 (15)	−0.0049 (9)	0.0041 (11)	−0.0085 (10)
C6	0.0842 (14)	0.0584 (12)	0.0646 (12)	−0.0103 (10)	−0.0065 (10)	−0.0105 (10)
C7	0.0635 (12)	0.0526 (11)	0.0546 (11)	−0.0049 (8)	−0.0053 (9)	0.0016 (8)
C8	0.0396 (9)	0.0439 (9)	0.0516 (10)	−0.0019 (7)	0.0007 (7)	0.0025 (7)
C9	0.0502 (10)	0.0433 (9)	0.0485 (10)	−0.0011 (7)	−0.0052 (7)	0.0089 (7)
C10	0.0589 (11)	0.0427 (10)	0.0562 (11)	−0.0010 (8)	−0.0035 (8)	0.0040 (8)
C11	0.0489 (10)	0.0431 (10)	0.0514 (10)	−0.0033 (7)	0.0032 (7)	0.0030 (7)
C12	0.0439 (10)	0.0472 (10)	0.0681 (12)	−0.0011 (7)	−0.0023 (8)	0.0004 (8)
C13	0.0534 (11)	0.0418 (10)	0.0706 (12)	0.0031 (8)	0.0033 (9)	0.0025 (8)
C14	0.0543 (11)	0.0434 (9)	0.0506 (10)	−0.0084 (7)	0.0036 (8)	0.0015 (7)
C15	0.0499 (10)	0.0553 (11)	0.0673 (12)	−0.0016 (8)	−0.0093 (9)	0.0009 (9)
C16	0.0559 (11)	0.0442 (10)	0.0718 (12)	0.0069 (8)	−0.0075 (9)	0.0004 (8)
C17	0.0664 (12)	0.0532 (11)	0.0626 (12)	−0.0073 (9)	−0.0030 (9)	0.0002 (9)

### Geometric parameters (Å, °)

**Table:**

O1—C1	1.3674 (19)	C7—H7	0.9300
O1—C10	1.4269 (19)	C8—C9	1.414 (2)
N1—C5	1.310 (2)	C9—H9	0.9300
N1—C4	1.369 (2)	C10—C11	1.503 (2)
N2—C17	1.140 (2)	C10—H10A	0.9700
C1—C9	1.363 (2)	C10—H10B	0.9700
C1—C2	1.410 (2)	C11—C12	1.381 (2)
C2—C3	1.358 (2)	C11—C16	1.386 (2)
C2—H2	0.9300	C12—C13	1.377 (2)
C3—C4	1.409 (2)	C12—H12	0.9300
C3—H3	0.9300	C13—C14	1.385 (2)
C4—C8	1.409 (2)	C13—H13	0.9300
C5—C6	1.398 (3)	C14—C15	1.389 (2)
C5—H5	0.9300	C14—C17	1.435 (2)
C6—C7	1.352 (3)	C15—C16	1.373 (2)
C6—H6	0.9300	C15—H15	0.9300
C7—C8	1.408 (2)	C16—H16	0.9300
			
C1—O1—C10	117.34 (12)	C1—C9—H9	120.1
C5—N1—C4	116.62 (16)	C8—C9—H9	120.1
C9—C1—O1	125.61 (15)	O1—C10—C11	108.09 (13)
C9—C1—C2	120.38 (15)	O1—C10—H10A	110.1
O1—C1—C2	114.01 (14)	C11—C10—H10A	110.1
C3—C2—C1	120.44 (16)	O1—C10—H10B	110.1
C3—C2—H2	119.8	C11—C10—H10B	110.1
C1—C2—H2	119.8	H10A—C10—H10B	108.4
C2—C3—C4	120.86 (16)	C12—C11—C16	118.59 (15)
C2—C3—H3	119.6	C12—C11—C10	120.62 (15)
C4—C3—H3	119.6	C16—C11—C10	120.74 (15)
N1—C4—C8	122.97 (16)	C13—C12—C11	121.32 (16)
N1—C4—C3	118.47 (15)	C13—C12—H12	119.3
C8—C4—C3	118.53 (15)	C11—C12—H12	119.3
N1—C5—C6	124.62 (18)	C12—C13—C14	119.39 (16)
N1—C5—H5	117.7	C12—C13—H13	120.3
C6—C5—H5	117.7	C14—C13—H13	120.3
C7—C6—C5	119.14 (18)	C13—C14—C15	120.02 (15)
C7—C6—H6	120.4	C13—C14—C17	120.32 (16)
C5—C6—H6	120.4	C15—C14—C17	119.67 (16)
C6—C7—C8	119.31 (17)	C16—C15—C14	119.63 (16)
C6—C7—H7	120.3	C16—C15—H15	120.2
C8—C7—H7	120.3	C14—C15—H15	120.2
C7—C8—C4	117.33 (15)	C15—C16—C11	121.04 (16)
C7—C8—C9	122.75 (15)	C15—C16—H16	119.5
C4—C8—C9	119.89 (15)	C11—C16—H16	119.5
C1—C9—C8	119.88 (15)	N2—C17—C14	179.4 (2)
			
C10—O1—C1—C9	−0.1 (2)	O1—C1—C9—C8	178.66 (15)
C10—O1—C1—C2	179.55 (14)	C2—C1—C9—C8	−1.0 (2)
C9—C1—C2—C3	1.7 (3)	C7—C8—C9—C1	178.09 (16)
O1—C1—C2—C3	−177.98 (16)	C4—C8—C9—C1	−0.1 (2)
C1—C2—C3—C4	−1.3 (3)	C1—O1—C10—C11	−171.97 (14)
C5—N1—C4—C8	−0.4 (3)	O1—C10—C11—C12	−135.85 (16)
C5—N1—C4—C3	177.42 (17)	O1—C10—C11—C16	46.7 (2)
C2—C3—C4—N1	−177.77 (16)	C16—C11—C12—C13	−0.6 (3)
C2—C3—C4—C8	0.2 (3)	C10—C11—C12—C13	−178.12 (16)
C4—N1—C5—C6	0.5 (3)	C11—C12—C13—C14	0.0 (3)
N1—C5—C6—C7	−0.1 (3)	C12—C13—C14—C15	0.7 (3)
C5—C6—C7—C8	−0.2 (3)	C12—C13—C14—C17	−179.84 (16)
C6—C7—C8—C4	0.3 (2)	C13—C14—C15—C16	−0.7 (3)
C6—C7—C8—C9	−178.00 (17)	C17—C14—C15—C16	179.81 (17)
N1—C4—C8—C7	0.1 (2)	C14—C15—C16—C11	0.1 (3)
C3—C4—C8—C7	−177.76 (16)	C12—C11—C16—C15	0.6 (3)
N1—C4—C8—C9	178.39 (15)	C10—C11—C16—C15	178.10 (16)
C3—C4—C8—C9	0.5 (2)		

### Hydrogen-bond geometry (Å, °)

**Table:**

*D*—H···*A*	*D*—H	H···*A*	*D*···*A*	*D*—H···*A*
C12—H12···Cg^i^	0.93	2.83	3.613 (2)	142
C13—H13···N1^ii^	0.93	2.60	3.398 (2)	145

Symmetry codes: (i) −*x*+1/2, *y*+1/2, −*z*+1/2; (ii) *x*, *y*+1, *z*.
